# Glucose excursions in type 2 diabetes modulate amyloid-related proteins associated with dementia

**DOI:** 10.1186/s12967-021-02797-3

**Published:** 2021-03-31

**Authors:** Abu Saleh Md Moin, Ahmed Al-Qaissi, Thozhukat Sathyapalan, Stephen L. Atkin, Alexandra E. Butler

**Affiliations:** 1 Diabetes Research Center (DRC), Qatar Biomedical Research Institute (QBRI), Hamad Bin Khalifa University (HBKU), Qatar Foundation (QF), PO Box 34110, Doha, Qatar; 2 Academic Endocrinology, Diabetes and Metabolism, Hull York Medical School, Hull, UK; 3 Leeds Medical School, Leeds, UK; 4 Royal College of Surgeons of Ireland, Busaiteen, Bahrain

**Keywords:** Type 2 diabetes, Glucose variability, Amyloid-associated proteins, Alzheimer’s disease

**To the Editor:**

We have reported that hypoglycemia was associated with changes in Alzheimer’s disease (AD)-related proteins [1] in accord with the direct link of cognitive dysfunction to hypoglycemia. Dementia is a recognized complication of type 2 diabetes (T2D) with an increasing prevalence, and AD is the most common cause. However, whilst the stress induced by hypoglycemia was likely the trigger, the contribution of the fall in glucose alone was unclear, given that glucose variability is associated with lower level cognitive function [2].

A case-controlled study was undertaken, enrolling type 2 diabetes (T2D) and control subjects. As detailed previously, each subject fasted for 10-h prior to undergoing a hyperinsulinemic clamp [3]. The mean baseline plasma glucose in the T2D cohort was 7.6 ± 0.4 mmol/l (136.8 ± 7.2 mg/dl); this was decreased to 4.5 ± 0.07 mmol/l (81 ± 1.2 mg/dl) for a duration of 1-h. For the control cohort, the mean plasma glucose was maintained at the baseline level of 4.9 ± 0.1 mmol/l (88.2 ± 1.8 mg/dl). To determine plasma levels of Alzheimer disease-related proteins, Slow Off-rate Modified Aptamer (SOMA)-scan measurement was undertaken, as previously detailed [3]. This analysis incorporated fifteen Alzheimer disease-related proteins: Serum amyloid A1 [SAA1], Alpha-synuclein [SNCA], Pappalysin [PAPPA], Noggin, Amyloid precursor protein [APP], Amyloid P component [APCS], Microtubule-associated protein tau [MAPT], Clusterin [CLU], Complement C3 (C3), Apolipoprotein A1 [ApoA1], Apolipoprotein B [ApoB], Apolipoprotein E [ApoE], Apolipoprotein E2 [ApoE2], Apolipoprotein E3 [ApoE3], Apolipoprotein E4 [ApoE4]. Graphpad Prism 8.0 was utilized to perform statistical analysis.

Age was comparable between T2D (n = 23) and control (n = 23) subjects (p = ns); as a group, the T2D cohort had an elevated BMI (p = 0.0012) with T2D disease duration 4.5 ± 2.9 years.

AD-related protein changes are shown in Fig. 1. APP was elevated at baseline (p < 0.01), and SNCA (p < 0.01) and ApoB (p < 0.05) decreased, in T2D whilst there was no difference between controls and T2D subjects at baseline for the other AD-related proteins. Following the hyperinsulinaemic clamp, APOA1 and C3 were significantly reduced (p = 0.05).

**Fig. 1:**
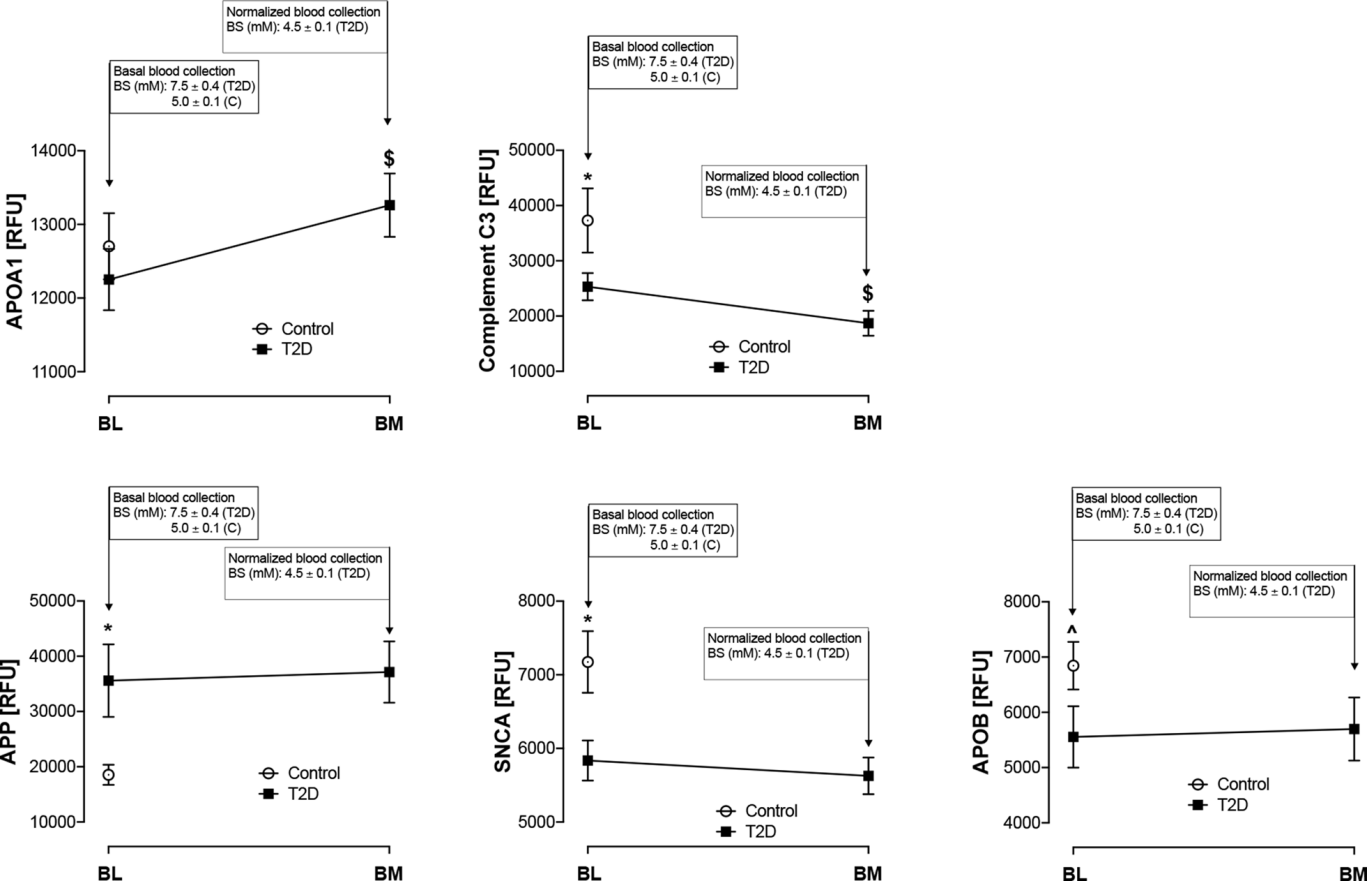
Circulatory levels of Alzheimer Disease (AD)-related proteins at baseline and upon glucose level normalization in T2D subjects. Blood sampling was performed at baseline (BL) in both controls (white circles) and T2D (black squares), and at glucose normalization (BM) in T2D subjects. At baseline (BL), blood sugar (BS) was 7.5 ± 0.4 mM (for T2D) and 5.0 ± 0.1 mM (for control, C). Proteomic (Somalogic) analysis of amyloid-related proteins was undertaken for fifteen amyloid-related proteins included in this analysis: Amyloid precursor protein [APP], Amyloid P component [APCS], Noggin, Alpha-synuclein [SNCA], Microtubule-associated protein tau [MAPT], Pappalysin [PAPPA] and Serum amyloid A1 [SAA1], Apolipoprotein A1 [ApoA1], Apolipoprotein B [ApoB], Apolipoprotein E [ApoE], Apolipoprotein E2 [ApoE2], Apolipoprotein E3 [ApoE3], Apolipoprotein E4 [ApoE4], Clusterin [CLU] and complement C3 (C3). Only proteins with changes at baseline between T2D and controls, or changes between BL and BM in T2D. Statistics: T2D vs control at BL: ^p < 0.05, *p < 0.01; T2D BL vs BM: $p < 0.05. RFU, relative fluorescent units

At baseline, the T2D cohort had higher circulating APP and lower SNCA; these results accorded with the findings in a previous report detailing circulating protein levels in AD [1]: accumulations of β-amyloid (Aβ) and tau proteins in the brain are pathognomonic of AD, and these deposits are believed to be a central facet in the pathophysiology of developing AD. It has been suggested that ApoB has a direct link to AD from analysis of rare coding variants of ApoB [4]. The protein component of HDL is comprised mainly of ApoA1; higher circulatory HDL/ApoA1 levels have been linked with decreased risk for AD and dementia [1]. Here, ApoA1 showed an increase with normoglycemia in T2D, but not in controls, suggesting that glucose normalisation was beneficial; future studies should study how glucose modulation affects ApoA1 levels. Conversely, C3 decreased with normoglycemia in T2D, but not controls. In the Copenhagen study of 95,442 individuals, lower complement C3 levels associated with higher risk for AD [5], suggesting that the fall in C3 with glucose normalisation may not be of benefit. ApoA1 concentrations have been positively correlated with circulating C3 [6], perhaps explaining why changes were seen for both, and their serum dysregulation has been reported in AD [6]. Glucose normalisation, falling by 55 mg/dl, occurred here and it is unclear whether the absolute glucose excursion or the rate of change of glucose were responsible for ApoA1 and C3 changes, and this requires clarification.

Study strengths include that T2D subjects had a relatively short duration of disease and were on minimal anti-diabetic therapy. Study limitations are the relatively small subject numbers and the small decrease in glucose for a short timeframe undertaken with the clamp.

In conclusion, AD-related proteins were lower in diabetes, and ApoA1 increased whilst C3 decreased significantly with euglycemia, suggesting that changes in these proteins may contribute to cognitive changes and this may be improved by good glucose control with reduction of glucose excursions.

## Data Availability

All the data for this study will be made available upon reasonable request to the corresponding author.
